# Monoclonal and oligoclonal TCR AV and BV gene usage in CD4^+^ T cells from pigs immunised with C-strain CSFV vaccine

**DOI:** 10.1038/s41598-018-19974-y

**Published:** 2018-01-26

**Authors:** Chunyan Wang, Shoujie Li, Huaijie Jia, Guohua Chen, Yongxiang Fang, Shuang Zeng, Xiaobing He, Wenjuan Yao, Qiwang Jin, Wenyu Cheng, Yuan Feng, Hong Yin, Zhizhong Jing

**Affiliations:** 1 State Key Laboratory of Veterinary Etiological Biology, Key Laboratory of Veterinary Public Health of Ministry of Agriculture, Lanzhou Veterinary Research Institute, Chinese Academy of Agricultural Sciences, Lanzhou, Gansu 730046 P.R. China; 2 Jiangsu Co-innovation Center for Prevention and Control of Important Animal Infectious Diseases and Zoonoses, Yangzhou, Jiangsu 225009 P.R. China

## Abstract

The classical swine fever virus C-strain vaccine (C-strain vaccine) plays a vital role in preventing and controlling the spread of classical swine fever (CSF). However, the protective mechanisms of C-strain vaccine and cellular immunity conferred by T cell receptors (TCRs) are less well defined. We aimed to analyse the association between the complementarity determining region 3 (CDR3) spectratype of αβTCR in CD4^+^ T cells and C-strain vaccine; and to find conserved CDR3 amino acid motifs in specific TCR α- and β-chains. We found that the CDR3 spectratype showed dynamic changes correlating with C-strain vaccine immunisation and that TCR AV5S/8–3S/8–4S/14/38 and BV4S/6S/7S/15S/30 gene families showed clonal expansion in immunised pigs. The sequences of CDR3 from these clonally expanded T cells indicated a high frequency of the ‘KLX’ motif in the TCR α chain and the ‘GGX’ motif in β chain, and Jα39, Jα43, Jβ2.5 and Jβ2.3 genes were also found in high frequency. To the best of our knowledge, this is the first report describing the dynamic changes of αβTCRs and conserved CDR3 amino acid motifs in CD4^+^ T cells from C-strain vaccine-immunised pigs, which will provide a basis for the development of high-efficiency epitope vaccines.

## Introduction

Classical swine fever (CSF) is a highly contagious disease that poses great risk to the swine industry worldwide, and it is characterised by fever, leucopenia, haemorrhage and high morbidity and mortality rates^1,2^. Its outbreaks often lead to severe economic losses worldwide. The causative agent is the CSF virus (CSFV), which belongs to the *Pestivirus* genus, *Flaviviridae* family^3^. At present, immunisation is used to prevent and control CSF. Live attenuated C-strain CSFV vaccines (C-strain vaccine) provide rapid onset and complete protection within 7 days, but immunological mechanisms that underlie the rapid protection afforded by C-strain vaccine are not well defined. Vaccination with C-strain vaccines can elicit neutralising antibody production and T cell responses. The vaccine can provide solid protection against virulent strain challenge at 7 days post immunisation (DPI)^4^ or even earlier^5–7^. However, neutralising antibodies, which are generally considered to be a major protective mechanism, cannot be detected until 2–3 weeks post immunisation^8^, indicating that cellular-mediated immunity induced by C-strain vaccine prior to this time is of great importance. In addition, in the absence of antibodies, virus-specific IFN-γ T cell responses can be detected at 7 DPI^9^; therefore, C-strain-conferred protection may still occur. Thus, virus-specific T cell responses may mediate protection under such circumstances^10,11^. Furthermore, C-strain vaccine appears to be able to stimulate CD4^+^ and CD8^+^ T cell responses, and the virus envelope glycoprotein E2 and non-structural viral protein NS3 have been described as targeted antigens^12–14^. Flow cytometric studies have revealed that virus-specific IFN-γ responses are predominant in CD4^+^ T cells and CD8^+^ cytotoxic T cells^15^. Therefore, understanding the immunological mechanism of rapid protection conferred by C-strain vaccine will be useful for developing the next effective vaccine.

T cells recognise specific antigenic peptides presented by major histocompatibility complex (MHC) molecules through the heterodimeric membrane protein T cell receptor (TCR)^16^. The complementarity determining region 3 (CDR3) of the TCR is a highly variable region responsible for recognising and interacting with various antigenic peptides^17^. Each CDR3 sequence refers to a specific T cell clone; thus, T cell clonality can be detected by monitoring the CDR3 spectratype^18^. As a more sensitive and accurate method, immunoscope spectratyping has been widely used to detect the clonality of T cells and to analyse the repertoire of TCR CDR3 genes^19^. The principle of this technique is to design specific forward TCR AV/BV primers for each family and conserved fluorescently-labelled reverse AC/BC primers. Scanning of fluorescent PCR products indicates the composition and expression frequency of each family. Under healthy conditions, TCRs show multi-family and multi-clonality characteristics; one gene family T cell contains different T cell clones, each with a different antigen-recognising ability. However, after responding to a specific peptide antigen, specific T cells proliferate and form clonal populations that react to the same peptide antigen^19,20^.

Previously, our laboratory has elucidated the CDR3 length repertoire and αβTCR gene diversity in porcine peripheral blood mononuclear cells (PBMCs)^21,22^ and CD4^+^ and CD8^+^ T cells under healthy conditions^23^. We also observed dynamic changes in the α- and β-chain variable regions of T cell receptor in the PBMCs of pigs infected by live attenuated C-strain CSFV by adaptation to culture in porcine kidney cells^21^. TCR analysis demonstrated monoclonal/oligoclonal expansion in the PBMCs of pigs infected with C-strain CSFV, and the sequencing of selected TCR CDR3 regions indicated a high level of conserved amino acid motifs^21^. Despite the demonstration of the relevance between clonally expanded TCR gene families and C-strain CSFV, the dynamics of the clonality of αβTCRs over time in CD4^+^ T cells from pigs immunised with C-strain vaccine and conserved CDR3 amino acid motifs remain unknown. In the present study, to characterise the immune status of C-strain vaccine-immunised pigs, we used reverse transcription polymerase chain reaction (RT-PCR) and GeneScan analysis to monitor TCR gene usage and clonal expansion in CD4^+^ T cells from four C-strain vaccine-immunised pigs and two non-immunised controls over time. The CDR3 regions of clonally-expanded TCR gene families were also sequenced. Our data may assist in elucidating the immunological mechanisms of the rapid protection conferred by C-strain vaccine and provide information on the design of new vaccine types.

## Results

### Detection of neutralising antibodies

Considerable anti-CSFV antibody titres were detected in all four immunised pigs, compared with the negative controls, but different antibody titres were observed at different times. An antibody titre of 1:16 was detected in pig H1 at 32 DPI, 1:256 in pig H2 at 7 DPI, 1:256 in pig H3 at 9 DPI and 1:32 in pig H4 at 24 DPI. In contrast, antibody titres of the negative controls were negative, which means that the four experimental pigs were successfully immunised, in spite of the great differences in the time points of antibody production and the levels among different pigs (Supplementary Figure 1 and Supplementary Table 2).

**Table 1:** Clonal expansions of αβTCR gene families in CD4^+^ T cells.

Source of T cells	TCR gene family	Case	Days post immunisation (DPI)		
			DPI = 3	DPI = 5	DPI = 7
CD4^+^ T cells	TCR AV	H1			
		H2	AV38^**#**^		AV14
		H3	AV8-3S^**#**^		
		H4	AV8-3S^**#**^, 8-4S, 14	AV5S^**#**^	
	TCR BV	H1			
		H2	BV6S^**#**^		
		H3	BV30		
		H4	BV4S, 7S, 15S		

^“#”^refers to the monoclonal expanded TCR gene families. Related to Fig. 3.

### CD4^+^ T cell clonality in C-strain vaccine-immunised pigs

**Figure 1:**
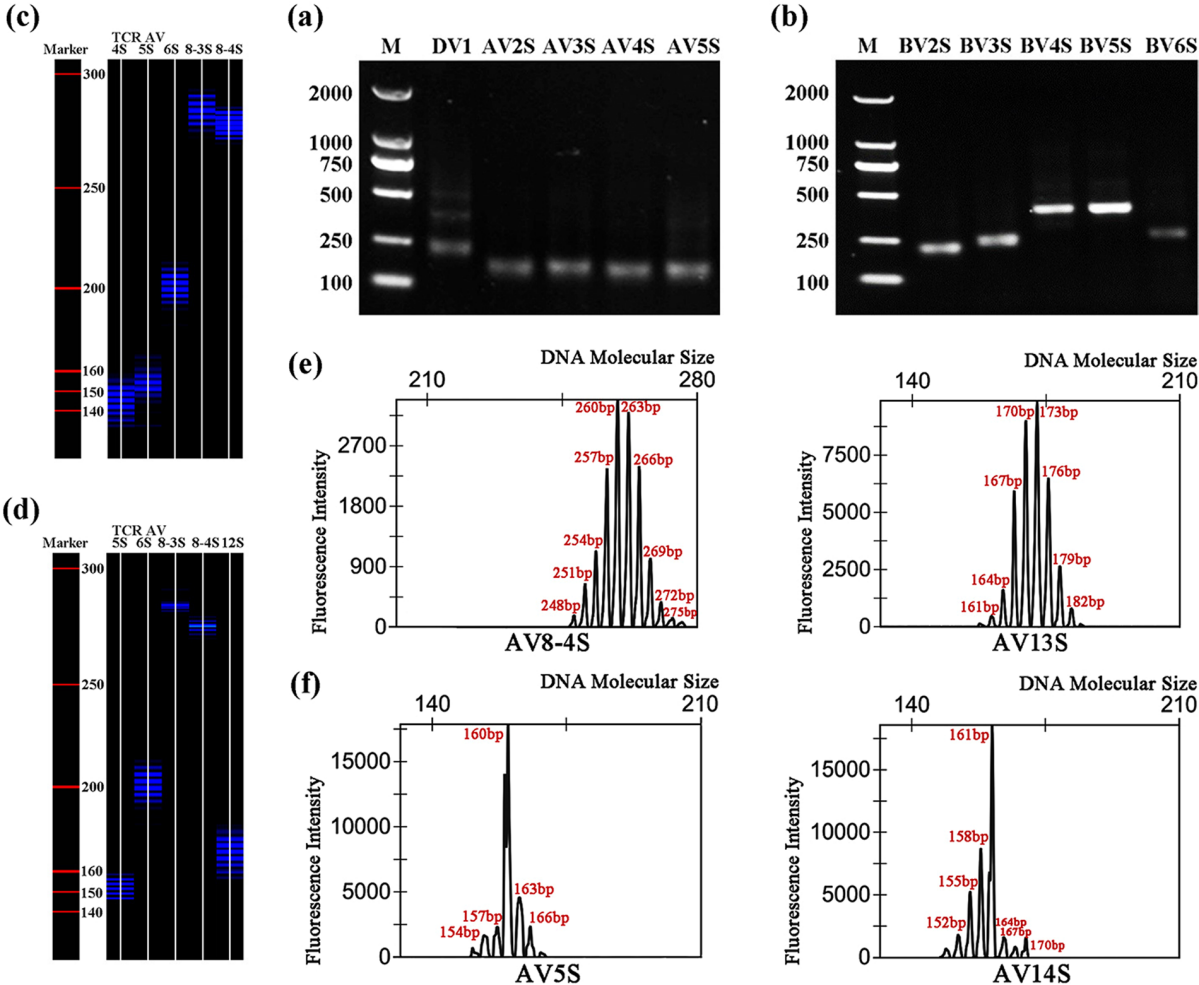
GeneScan results of partial TCR AV and BV gene families in CD4^+^ T cells from healthy control-1 (C1) and C-strain vaccine immunised pig-4 (H4). (**a**) The polymerase chain reaction (PCR) products of TCR DV1-AV5S of C1 analyzed on 1.5% agarose gel stained with ethidium bromide. (**b**) PCR products of TCR BV2S-BV6S of C1 analyzed on 1.5% agarose gel stained with ethidium bromide. (**c**) Partial fluorescence-labeled PCR products of C1 analyzed on 6% acrylamide sequencing gel. (**d**) Partial fluorescence-labeled PCR products of H4 analyzed on 6% acrylamide sequencing gel. (**e**) CDR3 size and fluorescence intensity analysis of TCR AV8-4S and AV13 S of C1. (**f**) CDR3 size and fluorescence intensity analysis of TCR AV14S and AV5S of H4.

**Figure 2:**
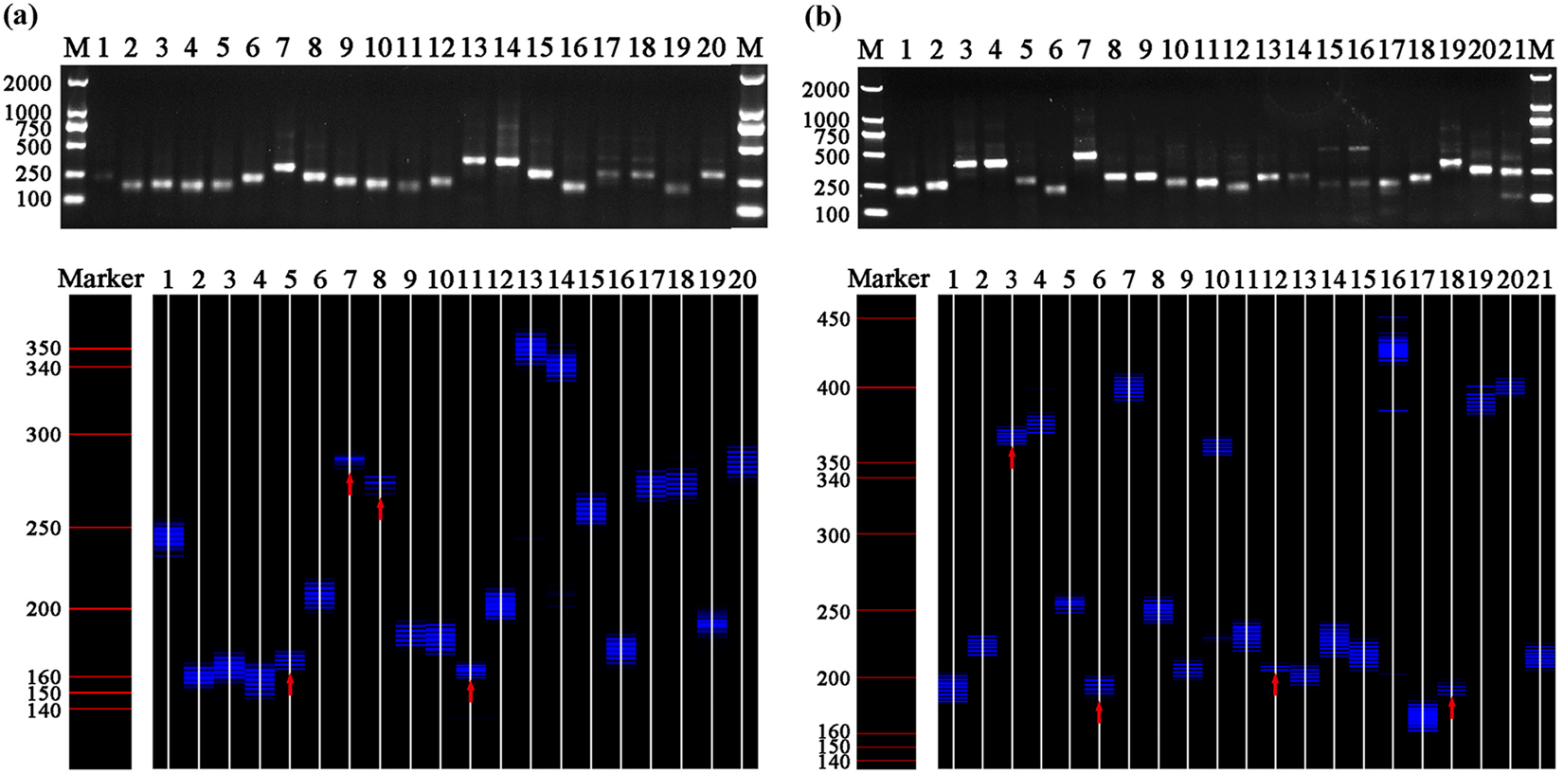
The patterns of CDR3 length distribution of 19 TCR AV and 20 TCR BV gene families in CD4^+^ T cells from C-strain vaccine immunised pig-4 (H4). (**a**) RT-PCR products of 19 TCR AV gene families in separated CD4^+^ T cells analyzed on 1.5% agarose gel stained with ethidium bromide and 6% acrylamide sequencing gel, respectively. (**b**) RT-PCR products of 20 TCR BV gene families in separated CD4^+^ T cells analyzed on 1.5% agarose gel stained with ethidium bromide and 6% acrylamide sequencing gel, respectively. Bands fewer than eight are marked with a red arrow.

Nearly every PCR product of the 19 TCR AV and 20 TCR BV gene families in the CD4^+^ T cells of healthy pigs showed a clear, specific and expected band size of approximately 250 base pairs (bp) on performing 1.5% agarose gel electrophoresis (Fig. 1a and b). However, these specific bands were obscure and could not even be seen in some TCR gene families of immunised pigs. The single band shown by agarose gel electrophoresis consisted of several bands with a 3 bp discrepancy between two adjacent bands, which could be separated in a sequencing gel. When visualised in a 6% acrylamide sequencing gel, more than eight bands could be seen in each TCR gene family in healthy control animals (Fig. 1c), while in the immunised animals, some TCR AV/BV gene families showed less than eight bands, with as few as two or three bands (Fig. 1d). For a representative pig (H4) post immunisation, the RT-PCR products of the 19 TCR AV and 20 TCR BV gene families were analysed on a 1.5% agarose gel stained with 10 μg/ml ethidium bromide and on an acrylamide sequencing gel by fluorescence (Fig. 2).

The CDR3 length distribution in the control and immunised pigs was also compared. In controls, the CDR3 peak map of each TCR gene family displayed a standard Gaussian profile, with at least eight peaks, and showed a symmetrical profile with the highest density in the middle and a 3-bp-gap in length between two adjacent CDR3 peaks. This signifies the high polymorphism of CDR3 and polyclonal T cell populations. AV8-4S and AV13S from one control pig (C1) were randomly selected to demonstrate this phenomenon (Fig. 1e). We also measured all other spectratypes of CDR3 and observed the same findings. By contrast, in the immunised pigs, some families showed abnormal CDR3 peak distribution of monoclonal/oligoclonal T cell expansion, with less than eight and as few as two or three peaks observed. AV5S and AV14S from pig H4 were randomly selected to demonstrate this biased distribution (Fig. 1f).

**Figure 3:**
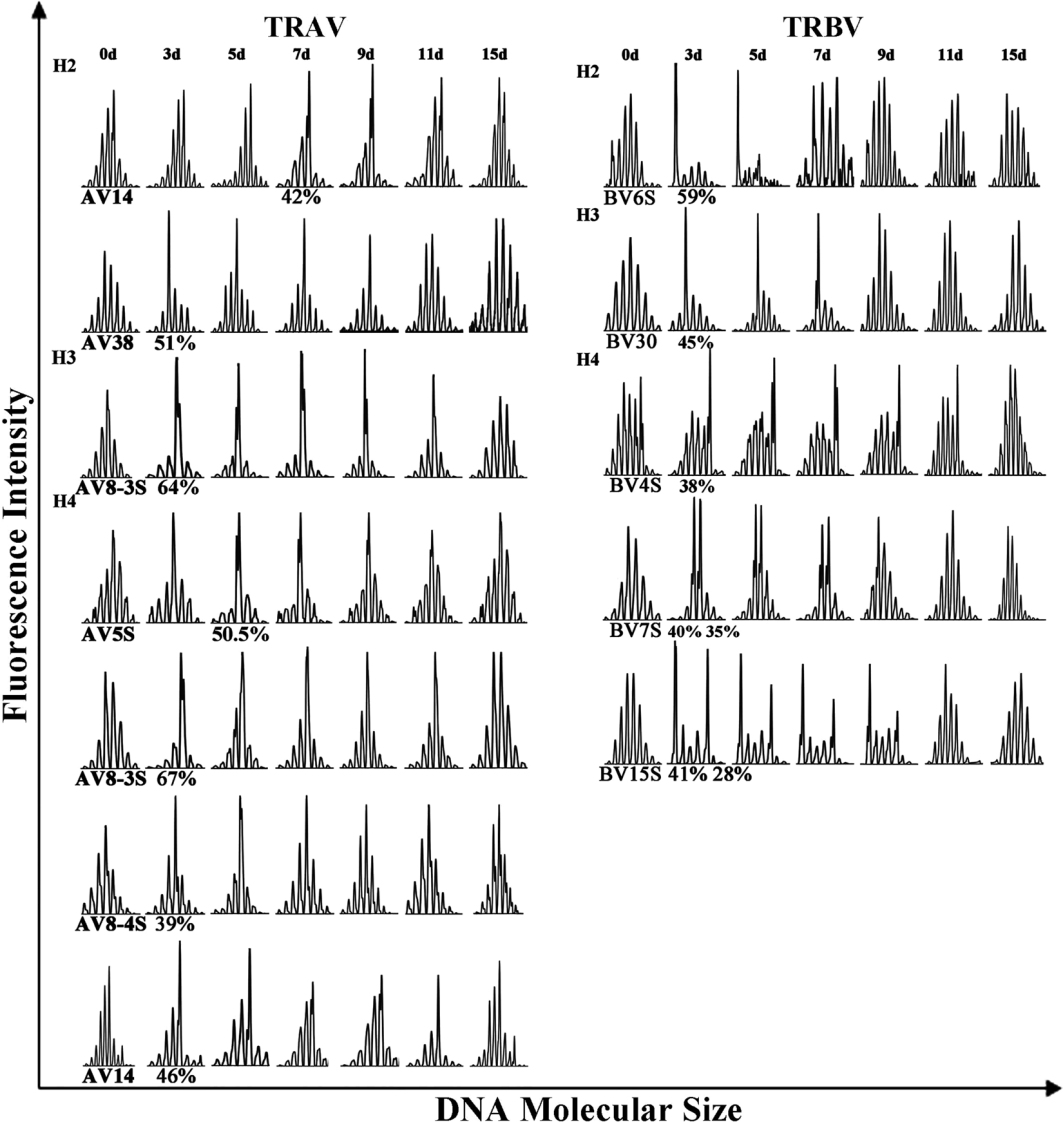
TCR AV and BV gene families showing evidence of clonal expansions in CD4^+^ T cells from C-strain vaccine-immunised pigs. The following criteria were used to determine whether a clonal T cell expansion had occurred: a single peak with an RI greater than 35%, twin peaks with each peak having an RI greater than 25%^19^. Peaks with an RI >50% of the total area were considered to be monoclonal expansions^24^. The RI value of each clonal expanded gene family were marked. Take pig H2 for example, TCR AV14 showed clonal expansion at 7 DPI, with an RI value of 42%. See also Table 1.

The RI value of each spectratyping peak was used to analyse clonal expansion in the CD4^+^ T cell populations in C-strain vaccine-immunised pigs. A single peak with an RI of >35%, twin peaks with each peak having an RI of >25% or a skewed family with a peak RI of >25% were considered to be clonally expanded T cells^19^. In addition, peaks with an RI >50% of the total area were considered to be indicative of monoclonal expansion^24^. According to this principle, the CDR3 distribution of CD4^+^ T cells showed different degrees of restriction. The number and type of V gene family with abnormal peaks varied between individual pigs (Fig. 3 and Table 1). Three pigs (H2, H3 and H4) showed clonally expanded TCR gene families but pig H1 did not.

**Figure 4:**
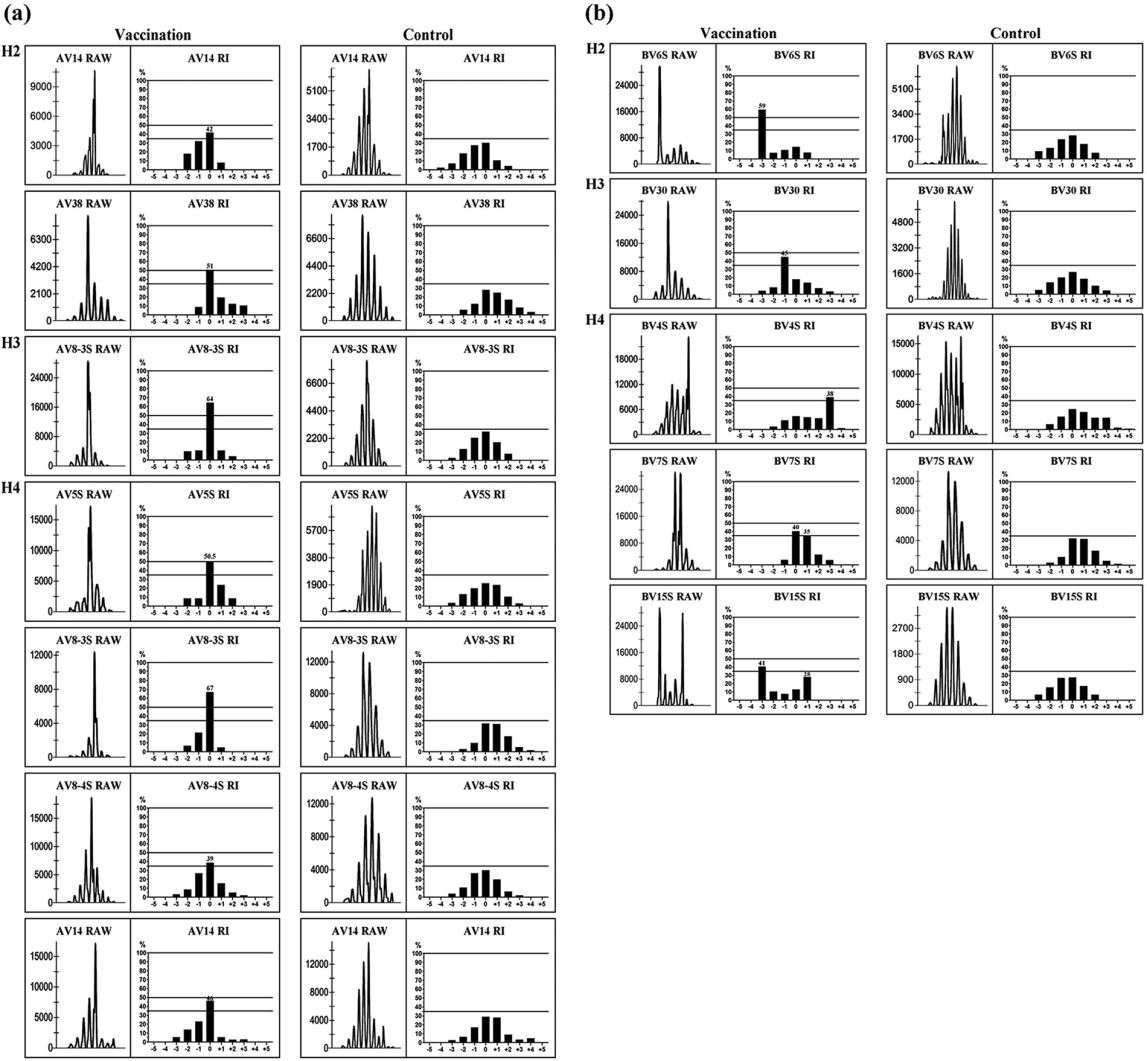
Clonal expansions of TCR AV and BV gene families in CD4^+^ T cells from pigs immunised with C-strain vaccine. Vα/β families with clonal expansions are depicted for post immunisation (left column) and before immunisation (recognized as control, right column). In each column, CDR3 size profiles and fluorescence intensity of selected families are shown on the left and RI value for each peak on the right. Numbers from −5 to +5 correspond to normally distributed CDR3 profiles from pigs before immunisation, where 0 is the major central peak. Data are shown as RI, calculated as each peak are against the total peak area. (**a**) TCR AV gene families showing evidence of clonal expansions. (**b**) TCR BV gene families showing evidence of clonal expansions.

In all pigs, 12 clonally expanded TCR gene families were detected in CD4^+^ T cells from C-strain vaccine-immunised pigs, corresponding to TCR AV14 (Peak 0), AV38 (0) and BV6S (−3) in H2; AV8-3S (0) and BV30 (−1) in H3 and AV5S (0), AV8-3S (0), AV8-4S (0), AV14 (0), BV4S (+3), BV7S (0, +1) and BV15S (−3, +1) in H4 (Fig. 4). The peaks were numbered according to their CDR3 length, compared with the major peak of each family in normal CD4^+^ T cells, which was given a value of zero. Five monoclonally expanded TCR families were detected in CD4^+^ T cells, corresponding to TCR AV38 (51%) and BV6S (59%) in H2; AV8-3S (64%) in H3 and AV5S (51%) and AV8-3S (67%) in H4. In addition, other clonally expanded TCR families were observed: AV14 (42%) in H2; BV30 (45%) in H3 and AV8-4S (39%), AV14 (46%), BV4S (38%), BV7S (40%) and BV15S (41%) in H4. Although CDR3 distribution showed a varied degree of restriction, TCR AV8-3S showed monoclonal expansion in both H3 and H4, while H2 and H4 displayed nearly the same TCR AV14 clonal expansion. In addition, compared with a previous analysis of clonal families in the PBMCs of C-strain vaccine-virus-challenged pigs^21^, we found that TCR AV5S, AV38 and BV6S not only showed clonal expansion in the PBMCs of C-strain vaccine-virus-challenged pigs but also displayed monoclonal expansion in the CD4^+^ T cells of C-strain vaccine-immunised animals.

### Monitoring of the CDR3 spectratype over time in the immunised pigs

We compared the CDR3 spectratype of each TCR gene family in the four experimental pigs before and at 3, 5, 7, 9, 11, 15 and 18 DPI. Some V gene families showing evidence of polyclonal expansion with normal Gaussian distribution before immunisation with C-strain vaccine changed into monoclonal/oligoclonal peaks at different times. This means that antigen-specific TCR gene rearrangement occurred due to vaccine stimulation, resulting in the preferential expression of several gene families and a decrease in TCR diversity. This kind of clonal expansion could be detected as early as 3 DPI in some families, including AV8-3S, AV8-4S, AV14, AV38, BV4S, BV6S, BV7S, BV15S and BV30. Still, other V gene families such as AV39 in pig H4 changed from being expressed at low levels to showing evidence of polyclonal expansion (Supplementary Figure 2). With time, the clonal phenomenon peaked and then returned to the pre-immunisation Gaussian distribution at 15 DPI (Fig. 3 and Supplementary Figure 2), which is consistent with that observed in previous studies^21^.

### Sequence analysis of the CDR3 region derived from normal spectratypes and clonally expanded TCR gene families

To determine whether there was expansion of particular clones, PCR products of CDR3 from those TCR gene families with normal Gaussian distribution and clonally expanded TCR AV and BV gene families were selected for sequencing. For the controls, we sequenced the CDR3 region of TCR AV5S (from H4) and BV30 (from H3) gene families, with normal spectratypes before immunization. Following cloning, 12 TCR clones in TCR AV5S and 8 in TCR BV30 were sequenced (Supplementary Table 3). Results showed that there were no identical sequences, indicating that TCR clones with normal Gaussian distribution were quite heterogeneous. The Jα/β gene also showed different usage. CDR3 from those TCR gene families that returned to Gaussian distribution at 15 DPI was also sequenced, and sequence diversity was similar to that before immunisation.

**Figure 5:**
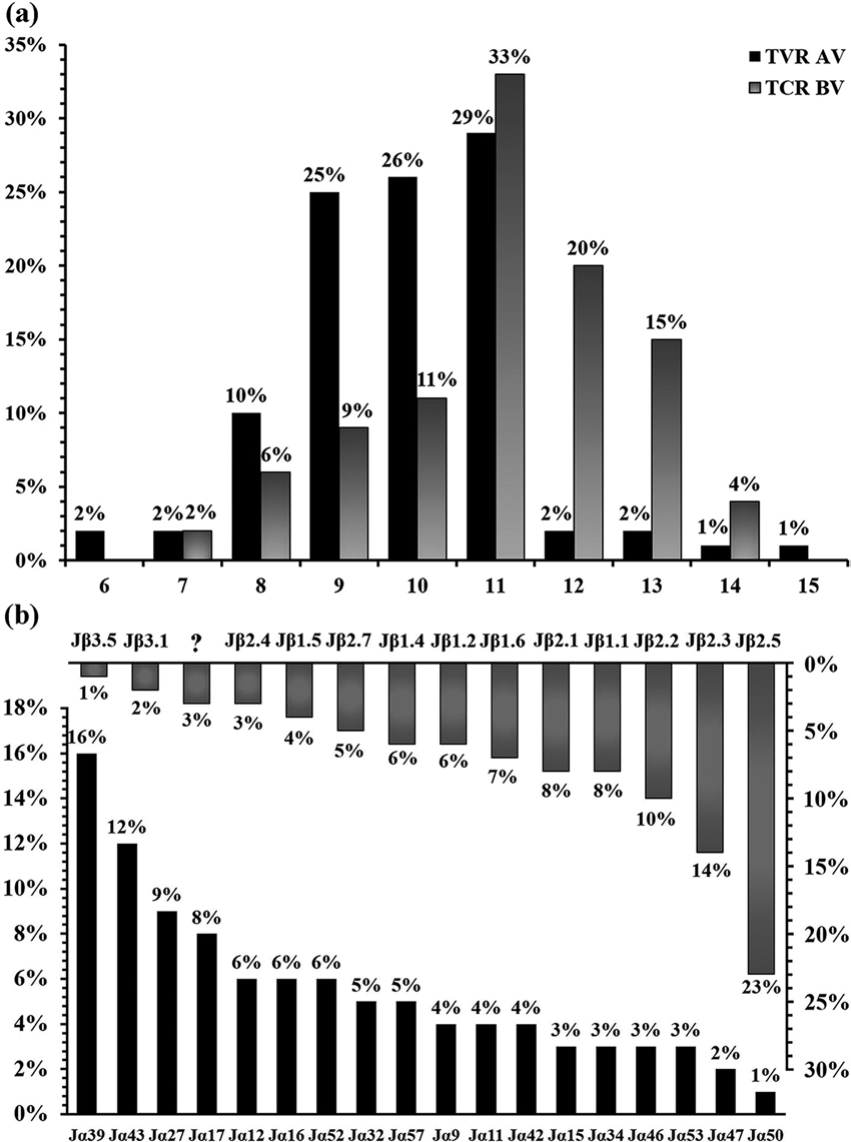
Analysis of the TRAV/BV CDR3 size selection and TRAJ/BJ gene usage by CD4^+^ T cells from C-strain vaccine immunised pigs. PCR products of CDR3 from clonally expanded TCR-α and -β chains were cloned and sequenced. (**a**) CDR3 size preferences by all sequenced Vα and Vβ genes from CD4^+^ T cells of C-strain immunised pigs are presented as percentage; 80% of clonally expanded T cells had a α CDR3 region of 9–11 aa in length, and preferred size was 29%; 68% of clonally expanded T cells had a β CDR3 region of 11–13, and preferred size was 33%. (**b**) The usage of TRAJ/BJ genes from clonally expanded TCR-α and -β chains in CD4^+^ T cells from pigs immunised by C-strain CSFV vaccine. “?” refers to the sequence that we didn’t find the corresponding TRBJ gene on GeneBank CoreNucleotide database (https://www.ncbi.nlm.nih.gov/genbank/) or IMGT website (http://www.imgt.org/).

The PCR products of CDR3 from clonally-expanded TCR AV and BV gene families were also cloned and sequenced. The TCR AV/BV CDR3 size and TCR AJ/BJ genes used by CD4^+^ T cells from C-strain vaccine-immunised pigs were analysed (Fig. 5). Results showed that 80% of clonally expanded T cells had an α-CDR3 region of 9–11 amino acids (aa) in length; and that the preferred size was 11 aa (29%). In a previous report of the CDR3 length in porcine TCR α-chain cDNA clones^25^, the average length was 9.35 aa and the preferred size was 8–10 aa. For TCR BV gene families, 68% of the clonally expanded T cells had a β-CDR3 region of 11–13 aa and the preferred size was 11 aa (33%). By contrast, the porcine TCR-β chain primarily has a CDR3 length of 36–45 nucleotides (12–15 aa)^22^. In TCR AV gene families, Jα39 and Jα43 showed a predominant usage of 28%; in TCR BV gene families, Jβ2.5 and Jβ2.3 had a high frequency of 37%. TCR BJ1-1, 1–2 and 3–7, which are located on the edge of the porcine TRBD-J-C clusters, showed a high frequency of usage when the expression of the detected TCR BJ segments was determined^26^. The amino acid sequences of CDR3 from those clonally-expanded gene families are summarised in Tables 2 and 3. It can be clearly seen that the predominant TCR clones account for 21–64%, indicating clonal expansion in particular T cells. The same or similar TCR AV/BV CDR3s could not be found in all clonally expanded gene families. However, the same or similar amino acid motifs were observed in some of them. For example, for TCR AV gene families (Table 2), AV38 of H2 and AV8-3S of H3 shared the same motif ‘NND’, AV38 of H2, AV8-3S of H3 and AV8-4S of H4 shared the same motif ‘GGX’. As a whole, nearly all clonal TCR AV gene families shared the same motif ‘KLX’. For TCR BV gene families (Table 3), BV30 of H3, BV4S of H4 and BV7S of H4 shared the same motif ‘GGX’; moreover, nearly all BV families shared the same ‘GX’ and ‘QX’ motifs.

**Table 2:** The CDR3 amino acid sequences of clonal TCR-α chain in CD4^+^ T cells in three Hezuo miniature pigs immunised with C-strain vaccine.

Case	TCR AV gene family	Peak	Size (aa)	3′ Vα	N + 5′ proximal Jα	Jα	Frequency
H2	AV14	0	11	CALS	ERDTRTD**KLT**FGDG	Jα27	3/14 (21.4%)
			11	CALS	ERRLSYD**KLI**FGSG	Jα34	2/14 (14.3%)
			11	CALS	ESRQTGYR**LV**FGKG	Jα15	2/14 (14.3%)
				Others			7/14 (50%)
	AV38	0	9	CAYS	AS**NNNDLR**FGAG	Jα43	3/11 (27.3%)
			9	CAYS	AT**NNNDLR**FGAG	Jα43	2/11 (18.2%)
			11	CAYS	AR**GGS**NY**KLT**FGKG	Jα53	2/11 (18.2%)
			10	CAYS	AQ**GGA**AGVFFGKG	Jα57	2/11 (18.2%)
				Others			2/11 (18.2%)
H3	AV8-3S	0	8	CAVS	FR**NNDLR**FGAG	Jα43	3/10 (30%)
			10	CAVS	G**NNGGF**KGVFGAG	Jα9	2/10 (20%)
			9	CAVS	AD**GGY**KWIFGRG	Jα12	2/10 (20%)
				Others			3/10 (30%)
H4	AV5S	0	11	CATG	VTISAGN**KLT**FGGG	Jα17	4/9 (44.4%)
			9	CATG	RGRTD**KLT**FGDG	Jα27	2/9 (22.2%)
			10	CATG	PDSGYS**KLT**FGKG	Jα11	2/9 (22.2%)
			8	CATG	STAN**KLV**FGPG	Jα32	1/9 (11.1%)
	AV8-3S	0	11	CALS	DLGAGYG**KLT**FGQG	Jα52	3/10 (30%)
			10	CAVS	PMYGAQ**KLV**FGRG	Jα16	3/10 (30%)
			11	CAVS	HSSGSGD**KLT**FGSG	Jα46	2/10 (20%)
				Others			2/10 (20%)
	AV8-4S	0	9	CALS	D**NAGKAFTF**GGG	Jα39	5/9 (55.6%)
			9	CALS	**GGS**QG**KLT**FGKG	Jα42	1/9 (11.1%)
			9	CALS	APSYN**KLM**FGQG	Jα50	1/9 (11.1%)
			9	CAVS	GSTAN**KLV**FGPG	Jα32	1/9 (11.1%)
			9	CALS	GNYGNKVIFGPG	Jα47	1/9 (11.1%)
	AV14	0	11	CALS	EPG**NAGKAFTF**GGG	Jα39	5/15 (33.3%)
			11	CALS	EPG**NACKAFTF**GGG	Jα39	2/15 (13.3%)
			11	CALS	EERGSQG**KLT**FGKG	Jα42	2/15 (13.3%)
				Others			6/15 (40%)

TRAV and TRAJ are assigned under the classification by IMGT website (http://www.imgt.org/). Numbers in parentheses are the frequency of this clone in the total cloned sequences.

**Table 3:** The CDR3 amino acid sequences of clonal TCR-β chain in CD4^+^ T cells in three Hezuo miniature pigs immunised with C-strain vaccine.

Case	TCR BV gene family	Peak	Size (aa)	3′ Vβ	N + Dβ + 5′ proximal Jβ	Jβ	Frequency
H2	BV6S	−3	11	CVVR	TLR**G**NAA**QL**YFGDG	Jβ2.2	7/11 (64%)
				Others			4/11 (36%)
H3	BV30	−1	12	CAWS	LA**GQGGH**QT**QY**FGPG	Jβ2.5	4/10 (40%)
			11	CAWS	LE**GRGGA**E**QH**FGPG	Jβ2.1	3/10 (30%0
			11	CASS	FM**GGG**P**QTQY**FGPG	Jβ2.5	1/10 (10%)
			13	CAWS	LRGTAASYAE**QH**FGPG	Jβ2.1	1/10 (10%)
			14	CAWS	RMSYGGGLT**QTQY**FGPG	Jβ2.5	1/10 (10%)
H4	BV4S	+3	13	CASS	L**GQGGA**MTDPLYFGEG	Jβ2.3	3/10 (30%)
			13	CASS	V**GQGGA**MTDPLYFGEG	Jβ2.3	2/10 (20%)
			13	CASS	SSH**GGA**VPDPLYFGEG	Jβ2.3	2/10 (10%)
				Others			3/10 (30%)
	BV7S	0	10	CASS	DWDRGKT**QY**FGPG	Jβ2.5	3/11 (27.3%)
			11	CASS	EA**GGL**S**QTQY**FGPG	Jβ2.5	3/11 (27.3%)
			10	CASS	IT**G**SDQA**QH**FGDG	Jβ1.5	2/11 (18.2%)
				Others			3/11 (27.3%)
	BV15S	−3	8	CASS	S**G**TIDYNFGPG	Jβ1.2	3/9 (33.3%)
			8	CASS	R**G**SNT**QH**FGPG	Jβ2.4	2/9 (22.2%)
				Others			4/9 (44.4%)

TRBV and TRBJ are assigned according to the reported sequences on GeneBank database. Numbers in parentheses are the frequency of this clone in the total cloned sequences.

## Discussion

The immunological mechanism induced by C-strain vaccine has not been well described, despite the effective rapid protection it confers. The earlier production of IFN-γ–specific T cells, compared with that of neutralising antibodies demonstrates that T cells may play critical roles in anti-CSFV immune responses, particularly prior to the production of neutralising antibodies. If this is the case, αβTCR repertoire diversity can be affected by CD4^+^ T cell clonal expansion following C-strain vaccine immunisation, resulting in decreased diversity and the selected usage of a limited number of TCR-α and -β chains, thus affecting the body’s immune response to this antigen. Based on this assumption, we attempted to identify CSFV-specific TCRs using the GeneScan spectratyping method. The degree of clonal proliferation and the functional status of T cells can be detected by monitoring the spectratypes, length or sequence of the CDR3 region of the TCR^27^. To the best of our knowledge, the present study is the first to describe the diversity and clonality of αβTCRs in CD4^+^ T cells from miniature pigs immunised with C-strain vaccine by immunoscope spectratyping. The data showed a diverse TCR repertoire under normal conditions, decreased TCR diversity post vaccination and monoclonally/oligoclonally expanded CD4^+^ T cells after C-strain vaccine immunisation. In addition, CDR3 from those TCR AV and BV gene families with clonal expansions demonstrated a high frequency of conserved amino acid motifs. The results of the present study revealed clonality changes in TCR gene families in CD4^+^ T lymphocytes from miniature pig immunised with C-strain vaccine.

TCR repertoire diversity is influenced by many factors. The likely contribution of TCRs specific to non-C-strain vaccine is unknown; therefore, it is critical to reduce other factors that may influence TCR repertoire diversity. We selected experimental animals that did not have any history of immunosuppressive diseases. Fluorescently-labelled PCR products of the CDR3 region of 19 TCR AV and 20 TCR BV gene families showed at least eight bands, with a 3 bp discrepancy between two adjacent bands on a 6% acrylamide sequencing gel, before immunisation. This is consistent with the hypothesis that under the condition of immune homeostasis, the spectratypes of CDR3 showed standard Gaussian distribution, indicating polyclonal expansion in T cells^28^. This kind of diversity of TCR is necessary for complete immunisation coverage. In contrast, less than eight bands, with as few as one or two bands, were observed among some PCR products of CD4^+^ T cells from the immunised pigs, which means that after responding to the specific antigenic peptide, some T cells clonally expanded. These clonal T cells had the same TCR that could react to the same antigenic peptide to induce cellular immune response. Spectratyping analysis showed that CDR3 peaks from several gene families evolved from standard Gaussian distribution peaks in normal pigs to oligoclonal distribution in immunised animals.

In an attempt to characterise TCR repertoire changes before and after C-strain vaccine immunisation, the CDR3 spectratypes were monitored over time, which is useful for identifying clonal expansion in CD4^+^ T cells. Clonal expansion in T cells resulted in a decrease in the peaks of the CDR3 region. We demonstrated that CD4^+^ T cells expanded following C-strain vaccine immunisation and that this was accompanied by the preferential expression of TCR AV5S, 8–3S, 8–4S, 14, 38 and BV4S, 6S, 7S, 15S and 30. This supports previous work that revealed the selective expression of TCR AV5S, AV38 and BV6S families in PBMCs following stimulation with C-strain vaccine-virus propagated in a PK-15 cell line^21^. However, in addition to the three TCR families mentioned in the above report, other gene families showing clonality were found in CD4^+^ T cells from vaccine-immunised pigs in the present study. This may be related to the difference in antigen loads between these two experiments because the TCR spectrum correlates negatively with the antigen load^29^ or constituents of the vaccine such as stabilisers. Further, different degrees of clonal expansion between individual pigs were observed, and there were differences in the type of clonal TCR gene families. Furthermore, several clonally expanded T cells in the TCR AV and BV gene families could be identified in the same pig. There is a similar phenomenon in case of clonally-expanded TRA and TRB subfamily T cells in peripheral blood from patients with diffuse large B-cell lymphoma (DLBL), without any particular correlation between specific TCR BV and anti-leukaemia T cell activities, reflecting the individuality of immune responses in different people^28^. In a study on systemic lupus erythematosus (SLE), Datta *et al*. found that autoimmune T cells in lupus patients can recognise five major epitopes in nucleosomal histones^30^. These clonally expanded T cells may recognise different CSFV antigens, or several major antigenic peptides of the same antigen may drive clonal T cell expansion in these C-strain vaccine-immunised pigs. To filter and confirm the major peptide-specific TCRs, we should simulate the possible TCR structure based on the particular TCR α- and β-chain CDR3 sequence in C-strain vaccine-immunised pigs and analyse the specificity of the TCR binding to major peptides using techniques including enzyme-linked immunospot assay (ELISPOT), fluorescence-activated cell sorting and tetramer assay^19^.

Although we have attempted to use age- and weight-matched genetically stable pigs and control feeding conditions, individual differences potentially leading to the differences in clonal TCRs are difficult to exclude. Clonally expanded T cells could be detected in pigs H2, H3 and H4, suggesting that the host immune response against CSFV could be detected by spectratyping analysis. However, there were no clonally expanded T cells observed in pig H1, despite the later production of neutralising antibodies. The precise reason for this is unknown. At present, studies on TCRs are mainly focused on human diseases and in mice, and there is little relevant research on TCR clonality in pig diseases. The oligoclonal activation of T cells in the PBMCs of C-strain vaccine-challenged pigs has been studied^21,22^. However, we still know little about CD4^+^ T cell clonality under vaccine-immunised conditions, such as how CDR3 spectratypes in CD4^+^ T cells and the clonotype of these clonally expanded T cells change. We compared CDR3 spectratypes from each TCR AV and BV gene family in four pigs over time and found that CDR3 spectratypes dynamically changed. Most clonally expanded TCR gene families can be detected early, at the 3 DPI, which is in accordance with previous report that after C-strain vaccination, the CSFV-specific T cell IFN-γ response can be detected in blood by as early as 6 or 8 days^31^. By contrast, antibody production in all four experimental animals was later than the appearance of clonal expanded T cells, which may indicate that T cell-mediated immune responses are likely to play an important role against classical swine fever, ahead of antibody production. However, clonal changes did not persist, but reverted to the standard Gaussian peak at 15 DPI. It has been reported that the proliferation of T cells reaches a peak after one or two weeks of exposure to antigen, after which T cells start to enter the stage of contraction. Most antigen-specific effector T cells show apoptosis, and only 5‒10% of cells survived as memory cells^32^.

Although Vα and Vβ family expansion profiles differed between pigs in our study, particular types of TCR families showed preferential usage more frequently than other gene families in C-strain vaccine immunised pigs, suggesting common antigen specificity. For example, the TCR AV14 family showed clonal expansion in pigs H2 and H4, while AV8-3S displayed clonality in pigs H3 and H4. In addition, three monoclonal gene families–TCR AV5S, 38 and BV6S were observed in the PBMCs of pigs inoculated with C-strain vaccine virus^21^. To better understand the shared nature of TCR gene families in CD4^+^ T cells associated with vaccine immunisation, CDR3 from gene families with normal Gaussian profile and from clonally-expanded T cells were sequenced. Among the 12 sequences of TCR AV5S and the 8 sequences of BV30 with normal spectratypes, there were no identical sequences, and the Jα/Jβ also showed different usage, indicating the multi-clonal expansion of T cells. For the CDR3 sequences derived from clonally expanded T cells, despite the sequence difference of different TCR genes and even the same TCR gene usage with different CDR3 sequences in different pigs, highly expressed amino acid motifs could easily be found. We found a highly conserved ‘KLX’ amino acid motif in nearly all TCR AV gene families, and a ‘GGX/QX’ motif in almost all TCR BV gene families. These results suggested that ‘KLX’ and ‘GGX’ may be linked to certain common CSFV antigen attributes and will facilitate the development of an epitope vaccine. In contrast, conserved motifs such as ‘DDYG’ and ‘FDDY’ in the CDR3 β-chain and ‘PAV’ in the CDR3 α-chain of PBMCs from pigs immunised with C-strain CSFV virus have been reported^21^. The sequence differences of the selected CDR3s revealed that CD4^+^ T cells and PBMCs may adopt different CDR3 motifs to recognise and react with a specific antigen. To produce either a protein vaccine or a DNA-based vaccine, protein structure modelling and the tetramer assay can be used to screen TCRs with the highest affinity for different types of SLA–peptide complexes. To confirm the conserved motifs and prove their association with C-strain vaccine, much higher numbers of pigs will be required in future studies.

The present study found that CD4^+^ T cells showed clonal expansion much earlier than neutralising antibody production, indicating that before antibodies play an anti-CSFV role, T cells are most likely to function as a key factor. Due to limitations in the number of experimental animals, our analysis may aid in understanding the molecular mechanisms of TCRs against C-strain vaccine, but more work is needed. A study including an additional cohort of animals would allow for the confirmation of data presented in our report. However, it is important to note that this is the first analysis regarding TCR clonality in CD4^+^ T cells from pigs immunised with C-strain vaccine, thereby providing useful information to understand anti-CSFV immune responses. In addition, public CDR3 characteristics of the TCR may be associated with certain common CSFV antigens. In human’s TCR research, M. tuberculosis 38-kDa antigen-specific TCRs were screened and expressed in TCR-gene modified CD4^+^ and CD8^+^ T cells, antigen-specific secretion of cytokine and cytolytic activity were seen in TCR gene modified CD4^+^ and CD8^+^ T cells^33^. In addition, antigen-associated monoclonal TCR Vα and Vβ gene families can be incorporated into antitumor or antiviral disease therapy^27,34^. Two patients with CD8^+^ T cells which were genetically engineered to express the melanoma MART-1-restricted TCRα/β gene win 18 months life time without this disease^35^. Whether such selected TCR genes in our study might be involved in the recognition of CSFV-associated antigens and play an effective role is important for future *in vitro* research. Our further studies will use an increased number of experimental animals to confirm conserved CDR3 amino acid motifs and clarify the relationship between conserved motifs and the corresponding antigen, which may lay a basis on the next development of epitope vaccine.

## Materials and Methods

### Ethical approval

This study was approved by the Animal Ethics Committee of the Lanzhou Veterinary Research Institute, Chinese Academy of Agricultural Sciences (Lanzhou, China; no. LVRIAEC2015–006). All animals were handled in accordance with the Animal Ethics Procedures and Guidelines of the People’s Republic of China. On predetermined days, all animals were euthanised by the intramuscular (i.m.) administration of ketamine-xylazine (Rompun) sedative, followed by intravenous administration of 5% sodium pentobarbital solution (100 mg/kg).

### Animal selection

A total of 6 healthy inbred 10-week-old Hezuo miniature pigs (3 females and 3 males), originating from the same litter at the State Key Laboratory of Veterinary Etiological Biology (Lanzhou, China), were used. None of the animals had ever been immunised with CSFV vaccines. The weights of the animals ranged from 12 kg to 15 kg. All animals were housed separately, with free access to food and water, and were kept on a 12-h-light/dark cycle at a temperature of 22 °C and humidity of 60%. All tested serologically negative for CSFV (cat. no. AP0000297), porcine reproductive and respiratory syndrome (cat. no. KQ0007), porcine circovirus (cat. no. K703213), porcine pseudorabies virus (cat. no. AP0000296), porcine parvovirus (cat. no. K703214) and foot and mouth disease virus (cat. no AP0001490), as determined using a serological detection kit (Wuhan Keqian Animal Biological Products Co., Ltd., Wuhan, China).

### Vaccination of pigs with C-strain vaccine

Prior to vaccination, 20 ml of heparinised peripheral blood was taken from all animals for T cell separation. Commercially available lyophilised live attenuated C-strain cellular vaccine (Weike Biotec, Harbin, China) was reconstituted with normal saline as directed, immediately prior to vaccination. Four miniature pigs (2 females and 2 males; designated as H1, H2, H3 and H4), classified as the experimental group, were intramuscularly immunised with four doses of 1 ml of reconstituted vaccine (750 PD_50_) per animal. Two negative controls (1 female and 1 male; designated as C1 and C2) received a similar immunisation of the same doses of normal saline.

### Isolation of sera

Fresh peripheral blood (2 ml) without any anticoagulant was collected on 3, 5, 7, 9, 11, 15, 18, 24 and 32 DPI, and serum was separated by centrifugation at 500 × *g* for 10 min.

### Neutralising antibody assay

CSFV neutralising antibody titres in sera were quantified according to the instructions for the indirect haemagglutination assay (IHA) kit made in the Lanzhou Veterinary Research Institute, Chinese Academy of Agricultural Sciences (Lanzhou, China). Briefly, sera were inactivated in a 56 °C water bath for 30 min. Two-fold serial diluted sera (including detected sera, positive and negative control sera) were added to 96-well plates, and this was followed by the haemagglutination antigen. According to the manufacturer’s instruction, antibody titres in sera were defined as the reciprocal of the highest dilution of the antibody at which hemagglutination inhibition was observed.

### Isolation of peripheral blood mononuclear cells (PBMCs)

Heparinised peripheral blood (20 ml) was taken from the precaval vein of the vaccinated pigs and control animals at 3, 5, 7, 9, 11, 15 and 18 DPI. Fresh peripheral blood was diluted with an equal amount of phosphate buffered saline (PBS) and was then slowly transferred to a 15-ml glass tube containing lymphocyte separation medium (Sigma-Aldrich; Merck KGaA, Darmstadt, Germany). The ratio of diluted peripheral blood to lymphocyte separation medium is 1:1. Following by horizontal gradient centrifugation at 400 × *g* for 20 min at 20 °C, PBMCs were separated and washed with cold PBS, and were then centrifuged at 450 × *g* for 10 min at 4 °C.

### Sorting of CD4^+^ T cells using magnetic beads

T cells were first enriched by nylon wool purification, following which indirect immunomagnetic positive sorting of CD4^+^ T cells was performed through the specific combination of phycoerythrin (PE)-conjugated anti-CD4 monoclonal antibody (BD Biotec, USA) and magnetic beads (Miltenyi Biotec GmbH, Bergisch Gladbach, Germany) labelled with anti-PE antibody, according to the manufacturer’s instructions. Following cell counting, T cells were incubated with PE-conjugated anti-CD4 monoclonal antibody for 20 min at 4 °C in the dark; a buffer (0.5% bull serum albumin and 2 mM EDTA in PBS, pH 7.2) was used to wash cells twice before they were incubated with microbeads. After incubation for 15 mins with microbeads at 4 °C in the dark, cells were then washed with the buffer and centrifuged at 300 × g for 10 min. The MiniMACS Column (Miltenyi Biotec GmbH) was placed in the magnetic field, and the column was washed with 500 µl buffer. Then, 1 × 10^8^ cells were loaded onto the column, and CD4^+^ T cells were separated according to the manufacturer’s protocol. To determine the purity of separated cells, separated cells were centrifuged at 300 × g for 5 min and washed twice with RPMI-1640 medium. Following counting, 1 × 10^6^ cells were resuspended in 100 μl FB (2% FCS and 0.1% sodium azide in PBS) and incubated with fluorescein isothiocyanate (FITC)-conjugated anti-CD3 monoclonal antibody (BD Biosciences, Franklin Lakes, NJ, USA) for 30 min. Cells were washed twice with cold FB and fixed in PBS containing 2% formaldehyde before they were subjected to flow cytometry analysis with a FACS Calibur flow cytometer (BD Biosciences); the results were analysed using BD CellQuest software (BD Biosciences).

### Extraction of RNA and synthesis of cDNA

Total RNA was extracted from 1 × 10^7^ sorted T cells using Trizol reagent (Invitrogen; Thermo Fisher Scientific, Inc., Waltham, MA, USA), according to the manufacturer’s instructions. RNA quality was determined by 1% agarose gel electrophoresis, which was stained using 10 μg/ml ethidium bromide. Total RNA concentrations were determined using a NanoDrop 2000 spectrophotometer (Thermo Fisher Scientific, Inc.), and the OD260:OD280 ratio of RNA was between 1.8 and 2.0. RQ1 RNase-Free DNase (Promega, USA) was used to degrade double and single-stranded DNA, according to the manufacturer’s instructions.

The first strand of cDNA was synthesised using the Primescript^TM^ 1^st^ strand cDNA synthesis kit (Takara Biotechnology Co., Ltd., Dalian, China), according to the manufacturer’s instructions, in a 20 μl reaction mixture. First, 1 μg RNA, 0.5 μl Oligo dT Primer (50 μM), 0.5 μl random hexamer primers (50 μM), 1 μl dNTP mixture (10 mM) and an appropriate volume of RNase-free water up to 10 μl were gently mixed, heated to 65 °C for 5 min; and then immediately chilled on ice. Next, the reverse transcription mixture was prepared: 4 μl 5× PrimeScript buffer, 0.5 μl RNase inhibitor (40 U/μl), 1 μl PrimeScript RTase (200 U/μl) and 4.5 μl RNase-free water were mixed with the aforementioned 10 μl mixture. The reaction mixture was incubated at 30 °C for 10 min, 42 °C for 50 min and 95 °C for 5 min to inactivate the RTase; and was then stored at −80 °C for the PCR amplification of all TCR gene families.

### Primers

Primers used for the specific amplification of 19 TCR AV families and 20 TCR BV families were synthesised based on previous research (Supplementary Table 1)^21,22^.

### PCR amplification of TCR α- and β-chains

PCR amplification of TCR α-chain CDR3s was conducted in a volume of 25 μl, containing 2 μl first-stand cDNA, 0.4 μl 5′ AV primer (100 μM), 0.4 μl 3′ FAM-AC primer (100 μM), 2.5 μl 10× Taq PCR buffer, 2 μl dNTP mixture (2.5 mM), 0.25 μl Taq DNA polymerase (Takara, Dalian China) and 17.45 μl diethylpyrocarbonate (DEPC)-treated water. Following initial denaturation step at 95 °C for 5 min, PCR was conducted with 35 cycles of denaturation at 94 °C for 50 s, annealing at 60 °C for 15 s and extension at 72 °C for 30 s, with final extension at 72 °C for 5 min. An aliquot of 8 μl of each PCR product was electrophoresed on a 1.5% agarose gel, stained with 10 μg/ml ethidium bromide, and analysed under ultraviolet light. PCR amplification of TCR β-chain CDR3s was performed as described above.

### GeneScan analysis of CDR3 spectratypes

An aliquot of 2 μl fluorescent PCR product was mixed with 2 μl formamide, 0.5 μl loading dye (25 mM ethylene diamine tetraacetic acid and 50 ng/ml blue dextran) and 0.5 μl GeneScan-500 TAMRA dye-labelled size standards (Applied Biosystems; Thermo Fisher Scientific, Inc.). The mixture was denatured at 95 °C for 3 min; and incubated on ice for 5 min; then, 2 μl was loaded onto a 6% acrylamide sequencing gel and the gel was run for 2 h in a 50-lane Applied Biosystems 373 A DNA sequencer (Applied Biosystems; Thermo Fisher Scientific, Inc.). Data were analysed using GeneMapper software (version, 4.1; Applied Biosystems; Thermo Fisher Scientific, Inc.)^22^. The evaluation of relative fluorescence intensity (RI) was described as follows: RI (%) = 100 × (clonal peak area)/(total peak area). The following criteria were used to determine whether clonal T cell expansion had occurred: a single peak with an RI of >35%, twin peaks with each peak having an RI of >25%, skewed distribution with the peak RI of the skewing family of >25% or skewed distribution with some families being expressed at very low levels or not at all^19^. Peaks with an RI of >50% of the total area were considered to be indicative of monoclonal expansion^24^.

### Determination of CDR3 nucleotide sequences

The PCR amplification mixtures of the TCR gene families showing standard Gaussian profiles and clonal expansion were selected for amplification in a final volume of 50 μl, containing 4 μl first-stand cDNA, 0.8 μl forward AV or BV primer (100 μM), 0.8 μl unlabelled reverse AC or BC primer (100 μM), 5 μl Taq PCR buffer (10 × ), 4 μl dNTP mixture (2.5 mM), 0.5 μl Taq DNA polymerase (Takara Biotechnology Co., Ltd.) and 34.9 μl DEPC-treated water. Primer sequences are listed in Supplementary Table 1. Thermal cycling parameters used were the same as those described for the PCR amplification of TCR AV and BV gene families. PCR products were electrophoresed on a 1.5% agarose gel, stained with 10 μg/ml ethidium bromide; and analysed under ultraviolet light. They were then purified using a gel extraction kit (Axygen; Corning Incorporated, Corning, NY, USA). Purified PCR products were ligated into the pGEM-T Easy vector (Promega Co., Madison, WI, USA) in a 16 °C water bath overnight, according to the manufacturer’s protocol. Following transformation into DH5α competent cells, a final concentration of 100 μg/ml ampicillin (Sigma-Aldrich; Merck KGaA, Darmstadt, Germany) was used to select positive clones. Nucleotide sequences were determined by Genescript Co., Ltd (Nanjing, China). The CDR3 region was defined as the region starting from the third amino acid after the last conserved cysteine (C) encoded in the V region and ending at the amino acid before the consensus FGXG box in the J region, as previously described^22,36^.

## Electronic supplementary material

Supplementary Information
