# Supplementary material for: Monoclonal and oligoclonal TCR AV and BV gene usage in CD4+ T cells from pigs immunised with C-strain CSFV vaccine
Source: Sci Rep. 2018 Jan 26;8:1655. doi: 10.1038/s41598-018-19974-y (PMC5786037; doi:10.1038/s41598-018-19974-y)
Supplement: Supplementary file 1 — Supplementary Information [file 41598_2018_19974_MOESM1_ESM.doc]

**Supplementary Information**

**Title: Monoclonal and oligoclonal TCR AV and BV gene usage in CD4^+^ T cells from pigs immunised with C-strain CSFV vaccine**

**Author list and Email address:**

| Chunyan Wang, wcy19900607@163.com | Shoujie Li, lishoujie2014@163.com |
| --- | --- |
| Huaijie Jia, huaijiejia@163.com | Guohua Chen, chenguohua78@163.com |
| Yongxiang Fang, yongxiangf@163.com | Shuang Zeng, zengshuang@caas.cn |
| Xiaobing He, hexb835@163.com | Wenjuan Yao, ywjdongyi@163.com |
| Qiwang Jin, jqw0902@163.com | Wenyu Cheng, wenyucheng1989@163.com |
| Yuan Feng, fengyuan19891203@163.com | Hong Yin, yinhong@caas.cn |
| Zhizhong Jing, zhizhongj@163.com |  |

**Correspondence to:** Dr Zhi-Zhong Jing, State Key Laboratory of Veterinary Etiological Biology, Key Laboratory of Veterinary Public Health of Ministry of Agriculture, Lanzhou Veterinary Research Institute, Chinese Academy of Agricultural Sciences, Lanzhou, Gansu, 730046, P.R. China.

E-mail: [zhizhongj@163.com](mailto:zhizhongj@163.com)


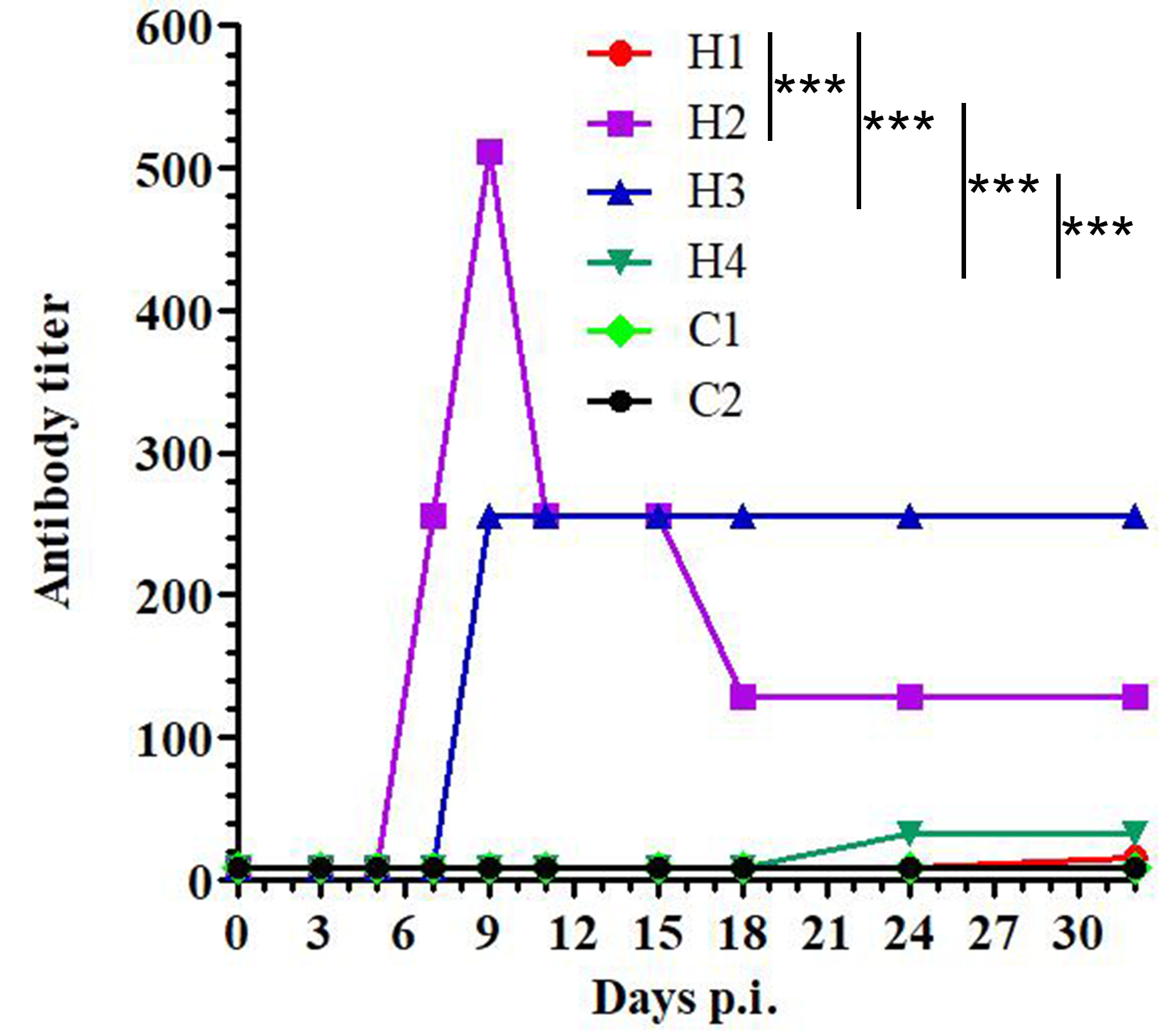


**Supplementary Figure 1**

The antibody titre in sera was defined by the reciprocal of the highest dilution of antibody at which inhibition of hemagglutination was observed. The data was analysed using SPSS software (SPSS 18.0 for Windows; SPSS, Chicago, IL, USA), with one-way analysis of variance followed by the Duncan’s multiple range test. ***P<0.001.


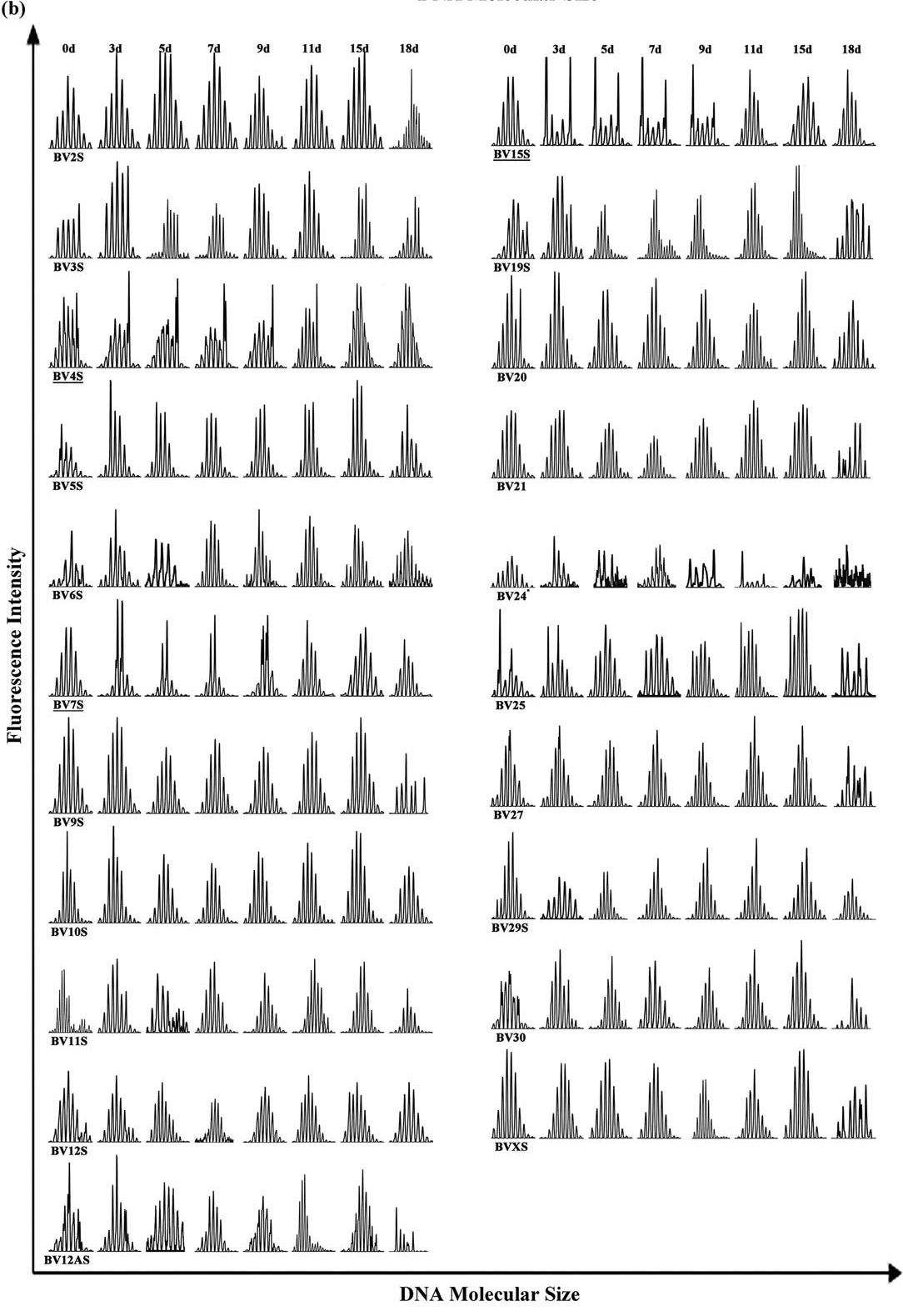

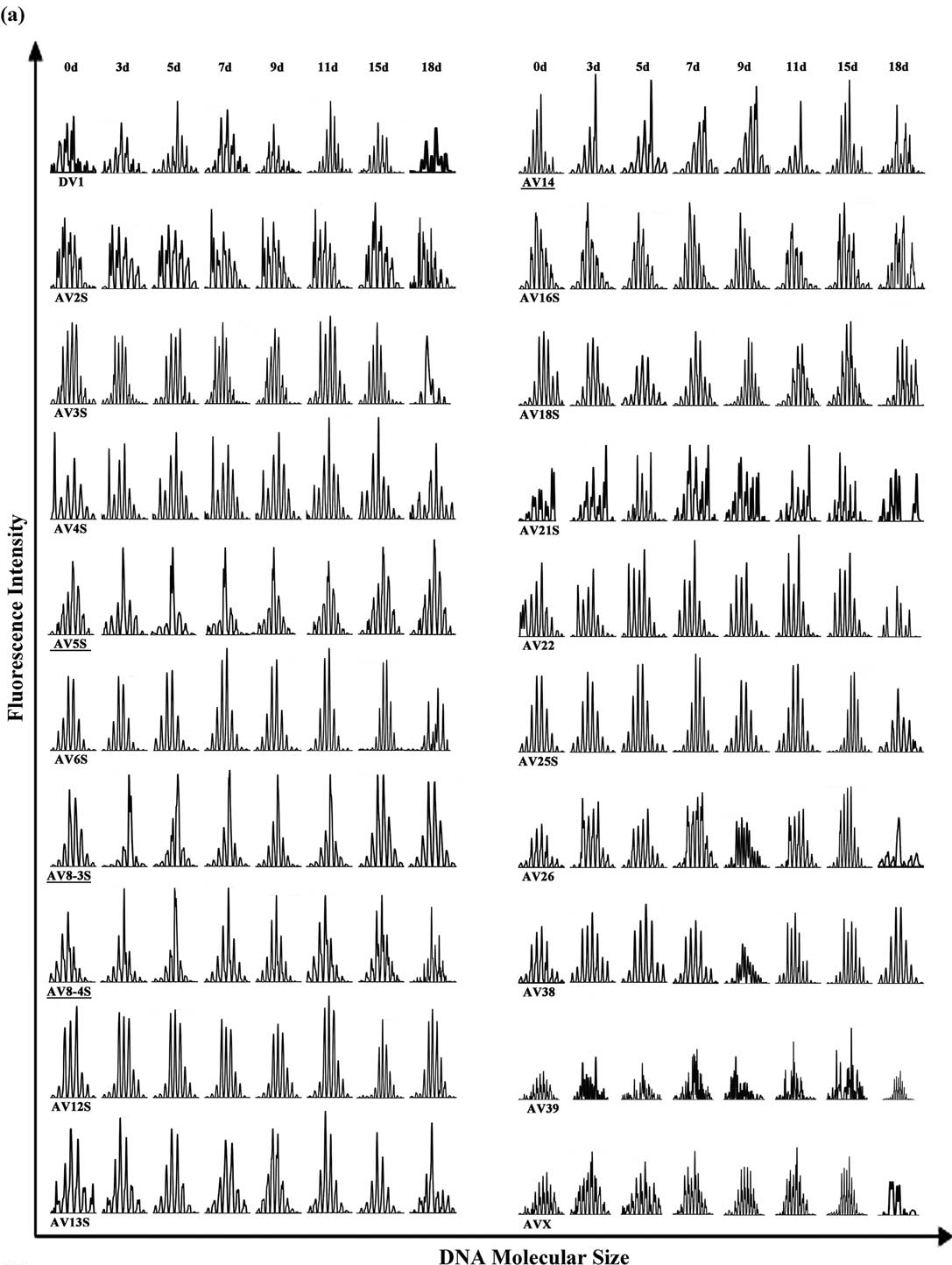


**Supplementary Figure 2. The whole CDR3 spectratype changes of 19 TCR AV and 20 TCR BV gene families in CD4^+^ T cells for one representative C-strain vaccine immunised pig-4 (H4) from before immunisation (0 d) to 18 days post immunisation (DPI).** The x-axis of each plot corresponds to the molecular size of the DNA, and the relative fluorescence intensity of the peaks is plotted on the y-axis. Gene families showing evidence of clonal expansion are underlined.

| TCR AV family | |  |  | TCR BV family | | |
| --- | --- | --- | --- | --- | --- | --- |
| Variable region | Primer sequence (5'-3') | Expected size (bp) |  | Variable region | Primer sequence (5'-3') | Expected size (bp) |
| DV1 | TGGCTGGAATGCAAAGGAAGA | 220 |  | BV2S | GGCACGTACCTGACTCTGAA | 190 |
| AV2S | TCAGGTGCAGGTGGCAGATG | 150 |  | BV3S | ACAGTTCCACGTCGCTTCTT | 220 |
| AV3S | CCAGCTGTCCTAGGGAGCGACT | 150 |  | BV4S | CAGATACCTGGTCCTGGGAA | 370 |
| AV4S | GGCCACCCTGAAAGACACTGC | 210 |  | BV5S | CACCGAGACATCTGATTAAAGC | 380 |
| AV5S | AAAATCACAGCAGCCCAACCTG | 153 |  | BV6S | TGGCATCACTGACAAAGGAG | 250 |
| AV6S | GAGCACCACCTTTGACACCAGAG | 200 |  | BV7S | TCTGAGCTGAAATTGCTCTCC | 190 |
| AV8-3S | TCCAGTACCCCAGCCAAGGA | 280 |  | BV9S | AGCTTTTGTCTCCACAGGTCA | 400 |
| AV8-4S | CAGAGGCTTTGGGGCTGAAT | 270 |  | BV10S | CCTGTGATGTTGGCATCCTT | 260 |
| AV12S | GCAAGCATGTCTCCCTGCTCA | 170 |  | BV11S | TGTTTCTCAGTTGCCCCAGA | 210 |
| AV13S | CTCCCTGCACATCGCAGTCA | 170 |  | BV12S | CACCCAGACACGAGGTGA | 340 |
| AV14 | TCTCAGATGCACAGGTGGAGGA | 160 |  | BV12A | CAACAACGGGTCTCCTGTG | 230 |
| AV16S | CCTCGACAAGAAAGAGGCATCC | 200 |  | BV15S | CGGCCTAACCCTTCTTTCTG | 210 |
| AV18S | TCTTCCAGAGGAGGCACCTATGAC | 350 |  | BV19S | CATTGACGCAGAAGAACCAG | 200 |
| AV21S | CGAGAGGGAGACGGCTTGGT | 340 |  | BV20 | ACAGCGCCAAGTTTCTCATC | 230 |
| AV22 | GGCGGCCTCATCAATCTGTTT | 250 |  | BV21 | ACAGCGATTTACAGCCGAGT | 210 |
| AV25S | GGACAGCTCCCTGCACATCA | 160 |  | BV24* | CTTTGTGGCCTTTTGCATCC | 420 |
| AV26 | TCGGCAAAATCCCAATCAGA | 280 |  | BV25 | CACCAGCCCTTCACAGACAT | 180 |
| AV38 | AGCTTCCCAACGGGGAGATG | 270 |  | BV27 | AGCCGAATTTCCCCTTGAT | 190 |
| AV39 | ACCAAAGCCCATTGCAGCAC | 180 |  | BV29 | ACCGTCAGCTTCTAGGACAAAG | 390 |
| AVX | TCGACAGTATCCAAATCAGGCACT | 280 |  | BV30 | TGACCAGAAAGATCCTGAAAAG | 400 |
| AC-FAM | TTTGGGGCCTTTCAGCTGGT |  |  | BVXS | ATCCCTTCCTGGAGCAGATT | 220 |
|  | | |  | BC-FAM |  |  |

**Supplementary Table 1. Primer sequences used for TCR AV/BV-specific amplifications.**

| Case | Days post immunization (DPI) | | | | | | | | | |
| --- | --- | --- | --- | --- | --- | --- | --- | --- | --- | --- |
|  | 0 | 3 | 5 | 7 | 9 | 11 | 15 | 18 | 24 | 32 |
| H1 | 8 | 8 | 8 | 8 | 8 | 8 | 8 | 8 | 8 | 16 |
| H2 | 8 | 8 | 8 | 256 | 512 | 256 | 256 | 128 | 128 | 128 |
| H3 | 8 | 8 | 8 | 8 | 256 | 256 | 256 | 256 | 256 | 256 |
| H4 | 8 | 8 | 8 | 8 | 8 | 8 | 8 | 8 | 32 | 32 |
| C1 | 8 | 8 | 8 | 8 | 8 | 8 | 8 | 8 | 8 | 8 |
| C2 | 8 | 8 | 8 | 8 | 8 | 8 | 8 | 8 | 8 | 8 |

**Supplementary Table 2. Antibody titers of pigs immunized with CSFV-C strain vaccine.** Experiments were performed at least two times. Related to Supplementary Figure 1.

|  | AV5S |  |  |  | BV30 |  |
| --- | --- | --- | --- | --- | --- | --- |
| 3′ Vα | N+5′ proximal Jα | Jα |  | 3′ Vβ | N+ Dβ+5′ proximal Jβ | Jβ |
| CATG | RYNQGGKLIFGQG | Jα23 |  | CAWS | LGHSYEQIFGPG | Jβ3.7 |
| CATG | LSGNNRFTFGKG | Jα7 |  | CAWS | PGWGGANRETQYFGPG | Jβ2.5 |
| CATG | RALSAGNKLTFGGG | Jα17 |  | CAWS | PGSGNYAEQHFGPG | Jβ2.1 |
| CATI | GGGFKVVFGAG | Jα9 |  | CAWS | PSVLWGANSNTEVFFGGG | Jβ1.1 |
| CAGN | FGGSQGKLTFGEG | Jα42 |  | CAWK | RSYGGGIYAEQHFGPG | Jβ2.1 |
| CAGA | GGSGRKPVFGTG | Jα44 |  | CAWV | DSGGSQTQYFGPG | Jβ2.5 |
| CAGA | RGSTANKLVFGPG | Jα32 |  | CAFR | DRLITDPLYFGEG | Jβ2.3 |
| CAGS | GNNDLRFGAG | Jα43 |  | CTCG | TAGASIQYFGPG | Jβ3.3 |
| CAGG | GGVLHFGSG | Jα35 |  |  |  |  |
| CAAS | GYSGVGSSQLTFGKG | Jα28 |  |  |  |  |
| CAAS | VTVNDLRFGAG | Jα43 |  |  |  |  |
| CAPV | VFSGVGSSQLTFGKG | Jα28 |  |  |  |  |

**Supplementary Table 3. The CDR3 amino acid sequences of AV5S and BV30 extracted from normal spectratypes.** TRAV/BV and TRAJ/BJ are assigned under the classification by IMGT website (<http://www.imgt.org/>) and GeneBank database, respectively.
